# Restorative Medicine

**Published:** 1872-01

**Authors:** 


*Restorative Medicine.* By Thomas King Chambers, M.D., etc. Philadelphia, Henry C. Lea, 1871.

"For upward of two centuries the London College of Physicians complied with the letter of Harvey's wishes, as expressed in his Deed of Gift, by causing an Annual Oration to delivered in Latin." This is the Harverian Annual Oration, and though delivered in England is published in America first. The address is ingenious and very instructive, and shows how our science, so-called, changes, and by what gradual steps the art of the old time is exchanged for the wholly unlike views of the present.

The "sequels" comprise two breakfast dialogues in which the question of Female Doctors, is discussed, and nearly all other questions. These chapters are highly instructive and amusing.

